# Creating breathing spaces for teenagers in the Welsh language: the case of theatre-in-Education

**DOI:** 10.1080/13569783.2023.2232314

**Published:** 2023-07-07

**Authors:** Hannah Sams

**Affiliations:** School of Culture and Communication, Swansea University, Swansea, United Kingdom

**Keywords:** Theatre-in-Education, Welsh, education, minority language

## Abstract

Despite the vibrancy and potential Welsh-language Theatre-in-Education (TIE) has thus far received very little scholarly attention. This article aims to address that gap in research by focusing on Welsh TIE's role in engaging teenagers with and through the Welsh language. The first part of this article outlines the socio-political, linguistic and educational context out of which TIE grew. The second part demonstrates how Welsh-language TIE responds to the Welsh context and has found ways of carving out spaces for teenagers to engage in the Welsh language beyond the classroom, providing a potential model for other minority language contexts.

## Introduction

Theatre-in-Education (hereinafter TIE) is a type of applied theatre that developed in England after the Second World War when political and educational ideals merged with a renewed focus on child-centred learning (Nicholson 2009, 36–39). TIE gained popularity in the 1960s: this was a period of rupture within the world of theatre in the United Kingdom, with many young theatre practitioners rejecting the values of both commercial theatre and traditional education (18). The aim of TIE was to encourage young people to participate in theatre as a learning medium and to think about social change (19). TIE was also seen as a means of democratising theatre as plays were often performed outside the traditional theatre setting, and were free at point of entry, making it a more accessible form for young people from a wide range of social backgrounds (Ogden 1997, 47). Unlike other forms of Children’s Theatre, TIE endeavoured ‘to achieve a change in understanding usually facilitated through participation, workshops and/or role play attached to a programme using a central theatrical stimulus’ (Wooster 2007, 46).

Although the first TIE companies were established in England, TIE proceeded to be ‘more strongly nurtured’ by the artistic establishment in Wales (3). Indeed, while English-language TIE companies in England and Wales have come and gone due to administrative and financial difficulties, it is the Welsh language and bilingual TIE companies that have managed to keep producing new material for young people in the educational system. These TIE companies have responded to changes in educational and artistic policy in Wales and created their own TIE model (Ogden 1997, 59).

Despite the resilience of these companies, their radical approaches and their cultural output, a limited amount of research has been undertaken on TIE in general and less still has been undertaken on the Welsh-language context (Jackson and Vine 2013; Jones 2014; Ogden 1997, 47–60; O’Toole 1976; Owen 2003, 116–191; Wooster 2007). Theatre for young people, especially theatre which is associated with the education system, is often underrated or simply ignored (Llewelyn 2009, 32; Turner 2009c, 49–51; van de Water 2013, 2). This article will situate the Welsh-language and bilingual TIE companies within the broader TIE movement and will proceed to provide a case study of one of the four existing Welsh-language TIE companies and its provision for teenagers specifically. In doing so, the article will show how Welsh-language TIE aimed at children and teenagers operate, the type of plays they produce, and how they interact with teenage Welsh speakers. It will argue that TIE is a unique approach in engaging teenagers within a minority language context and could be used in other bilingual educational environments.

Like many theatre companies working within minority and minoritised language contexts, Welsh-language theatre companies navigate a challenging linguistic environment. The most recent census data gathered in 2021 shows that the most dramatic reduction in Welsh speakers was amongst children and teenagers between 5 and 15 years old from 40.3% in 2011 to 34.3% in 2021 (Welsh Government 2022a). There is, therefore, an urgent need to engage with children and teenagers, two key age groups in terms of language maintenance (Gruffudd 1995; Welsh Government 2022b; Welsh Language Board 2006). Research has consistently shown (Gruffudd 2000; Morris 2010; Welsh Government 2022b) that the language of the home has the greatest influence on language use among youths, but 44.51% of the 39,516 young people attending Welsh-medium or bilingual secondary education come from non-Welsh speaking homes (Thomas 2022). Education is at the heart of the Welsh Governments’ Welsh language strategy which aims to more than double the number of Welsh speakers by 2050 as outlined in *Cymraeg 2050* (Welsh Government 2017, 2, 37–38). As well as having little or no contact with the Welsh language at home, the opportunities to use the Welsh language beyond the boundaries of the education system for the young people who live in communities where little, or no Welsh is spoken, are few and far between. Welsh-medium or bilingual schools are the main, and sometimes the only contact some young people have with the Welsh language. Despite the key role played by education in increasing the number of Welsh speakers, this does not always translate into language use in school or beyond the school gates (Price 2013, 253). Extracurricular activities, including theatre, therefore become even more important in contexts where Welsh is not the main community language (Fishman 1991, 317–373; Morris 2010, 96).

## Breathing spaces

This article will argue that Welsh-language TIE companies can act as what sociolinguist Joshua Fishman (1991) refers to as ‘breathing spaces’ (58–59) – physical spaces which are intentionally carved out to enable speakers of languages under threat to use that language free from the dominant language. The concept of breathing spaces was originally developed for speakers of languages under threat in urban areas and it is used in a variety of minority language contexts (Belmar and Glass 2019; O’Rourke 2019). Despite Welsh being the language of the classroom in Welsh-medium or bilingual schools, encouraging teenagers from non-Welsh speaking homes to speak Welsh beyond the classroom setting is still a challenge (Price 2013, 253). Teenagers who live in communities where little Welsh is spoken face further challenges as there are fewer opportunities to use the language within their communities (Baker 2006). This dependence on the education system to increase the number of speakers is common in minority language contexts, but sociolinguists such as Fishman (1991, 368–370) and Nicole Dołowy-Rybińska (2020, 113) have warned of the dangers of putting too much pressure on the education system to reverse language shift. This phenomenon isn’t exclusive to the Welsh-language context: a 2008 study by Comhairle na Gaelscolaíocht in Ireland showed that despite increasing the number of children and young people in immersion-language education programmes, social use of the language was still a matter of concern (Ó Riagáin and Moreno 2008). Welsh TIE performances and the schemes run by each of the companies to provide opportunities for children and teenagers to engage with the Welsh language are one way of creating breathing spaces for young people to engage with the Welsh language.

## History of the TIE

In 1965 the first professional TIE Company, Belgrade Theatre TIE Company, was established in Coventry. TIE was born out of a rejection of the values of both commercial theatre and traditional education, with the aim of encouraging both children and teenagers to participate in theatre as a learning medium and as a means of encouraging social change (Nicholson 2009, 19). Funded by the Coventry City Council, Belgrade Theatre provided a free at point of entry service, something that was at the core of its ethos. TIE was seen as a means of democratising theatre which was further facilitated by the fact that plays were often performed outside the traditional theatre setting, mainly in schools (Ogden 1997, 47). Many of the early TIE companies held socialist principles and saw TIE as a tool to empower audiences (Nicholson 2009, 19). In the early years especially, some of the defining characteristics of TIE were carefully devised performances aimed at small groups of children and teenagers (between 30 and 60 students) with a particular educational focus. The term ‘programme’ rather than ‘play’ was used to describe TIE work as it consisted of more than a one-off performance; the companies would often visit the school before the performance as well as offering a workshop after the performance with the aim of engaging the young audience with the piece. Audience participation was key: not only were the young audiences a part of the programme before and after the performance, but they were also encouraged to engage with the actors during the play and the young audience’s participation could influence the play’s outcome. This sort of theatre was devised and acted by specialist ‘actor-teachers’; a hybrid role specific to TIE groups.

The vision and the success of Belgrade Theatre was a source of inspiration for other TIE companies in urban areas such as Leeds, Bolton and Edinburgh. In the 1970s, at the height of TIE’s popularity in England, there were around 90 companies dedicated to creating work for children and young people in urban and rural areas alike, providing for all ages from infant school to further education colleges (Belgrade Theatre Company 2010; Jackson and Vine 2013, 26).

## History of the TIE movement in Wales

In 1971, 6 years after the establishment of Belgrade Theatre, Flint TIE became the first company dedicated to TIE in Wales (O’Toole 1976, 71). Several companies were formed in Wales shortly afterwards, including Breconshire TIE in 1971, which later became known first as the Breconshire Theatre Company and later renamed as Theatr Powys, a company described by Ogden (1997, 52) as ‘perhaps the most outstanding example of a TIE company in Wales’. Theatr Powys more than any other Welsh TIE company was more closely aligned with the work of Belgrade Theatre and other pioneers in the field. Despite their ambitious work for children and teenagers, like Spectacle Theatre company, Theatr Clwyd and Theatr Gwent, the Welsh language wasn’t used often in their productions. It is only when financial incentives were offered by the Welsh Office^1^ that things began to change (Owen 2003, 184).

In describing the vibrancy of TIE in Wales at the end of the 1980s, Ogden (1997, 51) notes that ‘there had come to exist in Wales a sustained provision of TIE in both Welsh and English that was greater in scope and vision than anything elsewhere in the UK’. TIE is considered one of the success stories of the Welsh professional theatre (Owen 2003, 167). Charmian Savill (1988, 49–54) in her survey of the TIE provision in Wales in 1988 noted that the provision ‘far outweighs that of its neighbouring countries Ireland, England and Scotland and is a leading example in European terms’. Both Roger Owen (2003, 167) and Matthew Jones (2014, 23) have made similar statements.^2^ TIE according to Roger Owen, more than any other form of theatre in Wales shows:

[ … ] how companies and audiences in Wales could assimilate an alien form of theatre and adapt it to their own requirements and purposes. This was done in Wales because a theatre in education company was in all eight counties that existed before local government was reorganised in 1994, and because those companies had carefully observed the inherent nature and needs of their own audiences. [my translation] (2003, 169–170)

The decision to link a TIE company with each of the (at the time) eight counties of Wales was taken by the drama officer at the Welsh Arts Council, Gilly Adams, in 1976, to ensure that all young people in Wales should have access to theatre. This proactive approach was another factor that aided the development of TIE in Wales.

## The evolution of TIE in the Welsh language

The context of political, linguistic and cultural discontent of the 1960s is integral to understanding what prompted the establishment of TIE companies in Wales and in the Welsh language. The political frustrations of the period such as the fight for more Welsh-language rights, Welsh road signs, Welsh education and a Welsh-language television channel, campaigns which were mostly led and initiated by university students in particular, are well documented (see for example Davies 2007, 545–615; Gruffudd 2000, 178; Llwyd 1986). In theatrical and cultural terms, young people felt frustrated by the tameness of the Welsh-language theatre scene, especially by Cwmni Theatr Cymru (1968–1982), that served as a national Welsh-language theatre. It was clear that the Welsh language was losing ground, and many felt that Cwmni Theatr Cymru and Welsh-language theatre in general were burying their heads in the sand by producing plays that failed to speak to Welsh people, especially young people, or engage with the political, cultural and linguistic issues at the time (Williams 2004, 261–263).

In response to the aforementioned political and cultural frustration, a number of new exciting and dynamic theatre companies were formed in the 1970s and 1980s, and there were several TIE companies among them. A new type of Welsh-language theatre emerged. The type of theatre created during the 1970s and 1980s has been described by Ioan Williams (263) as ‘flexible and mobile, focussing attention on the here and now of the performer, rather than on the fictionalised other space of dramatic literature. It offered, consequently, a mechanism for the generation of new audiences and new cultural experience’. The theatrical language of many of these companies, TIE included, was another means of protesting against the individualistic politics of the time. As Jeremy Turner, the current artistic director of Cwmni Theatr Arad Goch (hereinafter, Arad Goch), notes when describing the vibrancy of the scene in the 1970s and 1980s:

It was buzzing at the time, because we were young, fresh and we drew on the spirit of young people. Young people were trying to find new ways of doing theatre. There was a lot of energy, a lot of enthusiasm. And our aim was to create good theatre for young people, not pantomime. [my translation] (Tudur and Turner 2005, 21)

It was in this ‘buzzing’ context that the first Welsh-language TIE company emerged: Cwmni Theatr y Werin, Aberystwyth (1973–1981). Prior to this, only a few Welsh-language TIE productions had been attempted: Flint TIE produced *Syrcas Sulwen* (1972), a play aimed at children which John O’Toole refers to in his study of British TIE as a pioneering attempt in using theatre as a medium to teach a second language (O’Toole 1976, 71). Theatr Powys, first known as the Breconshire Theatre company before changing their name, staged a few Welsh-language plays, financed by the Welsh Office, the department within the Government of the United Kingdom responsible for Government policy in Wales between 1965 and 1999, the year in which Wales became a devolved political entity within the United Kingdom.

Although Cwmni Theatr y Werin was short-lived, another TIE company, Cwmni Theatr Crwban (1979–1989), was established in Aberystwyth to fill the void left by them. This was followed by Cwmni Cyfri Tri (1980–1989), formed by Aberystwyth University graduates, which was an experimental theatre company that decided to produce work for young people in its latter years. Due to the alignment of both Cwmni Theatr Cyfri Tri and Cwmni Theatr Crwban’s values and aims, as well as their geographical proximity, the companies merged in 1989 to form Arad Goch.

By the end of the 1990s, there were 8 TIE companies in Wales which served each of the 8 counties of Wales. The companies provided dynamic and exciting theatre programmes rooted in their communities, in the curriculum and in most cases in the Welsh language, with children and teenagers at the heart of everything they did. They performed in schools, theatres and village halls, and as well as providing theatrical experiences for children and teenagers, several TIE companies provided young actors, writers and theatre practitioners with opportunities to develop their craft (Table 1).

**Table 1.** List of 8 TIE companies.^a^

Company name	Company’s location
Cwmni Theatr Powys (1972–2011)	Powys, mid Wales
Cwmni Theatr Gwent (1976–2011)	Gwent, southeast Wales
**Cwmni Theatr na nÓg formerly known as West Glamorgan Arts (1981-)**^b^	Neath, south Wales.
**Cwmni Theatr y Frân Wen (1984-)**	Bangor, north Wales
**Theatr Iolo (1987-)**	Cardiff, south Wales
**Cwmni Theatr Arad Goch (1989-)**	Aberystwyth, mid Wales
Spectacle Theatre Company (1979-)^c^	Tonypandy, south Wales
Cwmni Theatr Clwyd (1976-)^d^	Mold, northeast Wales

^a^ Companies in bold are still in existence today.

^b^ Open Cast Theatre Company was one of the original companies. Open Cast Theatre closed its doors in 1979. West Glamorgan County Council and the Arts Council then founded TiC (Theatre in the Community). This morphed into Theatr Gorllwein Morgannwg under the leadership of Tim Baker and then to Thear na nÓg.

^c^ Spectacle Theatre still exists but since the funding review in 2011, it no longer receives funding from the Arts Council of Wales.

^d^ Theatr Clwyd is still active, although the TIE branch of the company is not active.

## Education in theatre

TIE, according to Anthony Jackson, is both vital and vulnerable due to its dependence on government and local authority funding and as a result it has had to face periods of cutbacks and uncertainty followed by periods of resurgence and recovery (Jackson and Vine 2013, 21). The Welsh TIE companies are certainly no exception. Considering TIE’s dependence on both Government funding and the education system, it is hardly surprising that changes to either impact their development and activity. One such change was the Educational Reform Act of 1988 followed by the implementation of the National Curriculum in England and Wales in 1989. The implementation of the curriculum in 1989 had a profound impact on Welsh-medium education and the development of a specific curriculum for Wales. For the first time in the history of Welsh education, the Welsh language became a compulsory subject for pupils aged between three to sixteen in Wales (James and Wynn 2003, 85–102; Jones and Roderick 2003, 198–234). In the early days, the Welsh language component was one of the few differences between the curricula in Wales and England. With the arrival of devolution, the educational provision for Wales has become more bespoke. Renaming the curriculum the Cwricwlwm Cymreig [Welsh Curriculum] in 2000 was a clear demonstration of division between the English and Welsh education systems (National Assembly for Wales 2001, 3; Williams 1997, 165).

The 1988 Education Reform Act involved more subject-centred teaching, with an emphasis on reaching a set of prescriptive guidelines with little cross-curricular activity. As well as changes to the curriculum, there were significant administrative and financial implications of the Educational Reform Act that posed a threat to TIE. Under the new Act, school finances were put in the hands of the schools themselves rather than the Local Education Authority, which had previously overseen school finances. It was the TIE companies that now had to compete with other pressing matters such as staff and book budgets. This change forced many companies to change their model by performing to larger groups of young people with fewer or no participatory workshops, a key identifying feature of the early form of TIE (Wooster 2007, 80.) Several companies, especially those in Wales, did manage to adapt, but some were forced to close their doors and the first professional company in England, Belgrade TIE, was a notable loss to the movement (Belgrade Theatre Company 2010, 15).

Despite having to face many of the same funding challenges as English TIE companies, the Welsh TIE still in existence today managed to overcome the challenges by responding to the curriculum and tailoring the TIE model to suit the needs and requirements of Wales and its educational and linguistic context, including what former actor/teacher and academic Roger Wooster (2007, 49) described as the ‘issue’ of the Welsh language. This forced many of the companies to depart from what some, such as O’Toole (1976, vii), consider to be the defining features of TIE: performing to small groups led by actor-teachers as well as the participatory element.

Further changes were prompted in 2010 after an Investment Review by the Arts Council of Wales (as quoted in Jones 2014, 25). Following the review, the funding was cut to three companies working predominantly through the medium of English: Cwmni Theatr Powys, Theatr Gwent and Spectacle Theatre. Although Theatr Clwyd is still an active theatre company, the company’s outreach work is no longer its central focus. The 2010 review stated that theatrical work which satisfied the didactic requirements of the curriculum would no longer be funded (26). Although Arad Goch, for example, did not restrict themselves to the term TIE, as Turner stated in an interview with Wooster (2007), Arad Goch’s emphasis is on ‘the “theatre” in “Theatre in Education”’ (80). The review did however lead several companies to explicitly rebrand themselves as ‘Theatre for Children Young Audiences’ or ‘Educational Theatre’. The model had to change with less emphasis on the participatory element of TIE and performing to such small groups would no longer be viable. Several of the current Welsh TIE companies now refrain from using the term TIE due to changes in funding priorities, preferring labels such as ‘theatre for children and young audiences’ or theatre to ‘ignite young imaginations’ (Nicholson 2009, 46–47; Theatr Iolo n.d.; Theatr na nÓg n.d.). Nicholson also discusses the change in terminology. Nicholson favours ‘educational theatre’. She sees this shift and willingness to use terms ‘theatre in education’ and ‘educational theatre’ or ‘creative learning’ to name but a few of the terms used as pertinent as it ‘indicates that a new generation of theatre-makers are eroding old divisions between theatre as an art form and theatre as a learning medium, between theatre-in-education and the theatre, between play and art’ (46–47). Despite the change in terminology, the essence of the work is the same – providing high quality theatrical experiences which inspire, educate and engage young audiences.

Adaption has been key to survival of the Welsh companies, and by adapting they re-emerge in ‘different shapes and more varied and more ﬂuid permutations’ (Jackson and Vine 2013, 17). Despite the adaptions and the change in the participatory nature of each company’s work, participation and ‘learning *through* theatre’ is still at the heart of it (Nicholson 2009, 38–39). Current Welsh theatre for children and young people maintain the participatory component of their work by offering work packages, language workshops and a whole host of other initiatives discussed later in this article to ensure creative engagement with the curriculum through the medium of theatre in both Welsh and English.

Another clear demonstration of how devolution has enabled Welsh policy makers to develop a curriculum with the children and young people of Wales specifically in mind was seen in 2022 with the introduction of the Cwricwlwm i Gymru/Curriculum for Wales (Welsh Government 2019). The radical curriculum reform in Wales for all learners aged between 3 and 16 focuses on 6 areas of learning, ‘Languages, Literacy and Communication’ and the ‘Expressive Arts’ being two of them. These areas of learning replace named subjects and associated assessments which according to Graham Donaldson, caused ‘unhelpful polarisation’ (Donaldson 2015, 36).

The Curriculum for Wales’ continuum-based approach to the teaching of Welsh aims to change the way Welsh is taught, and even more crucially *used* in schools. Welsh will be a more prominent part of children and teenagers’ education in Wales not only in Welsh-medium schools but also in bilingual and predominantly English-medium schools (Welsh Government 2017, 38).^3^

The Curriculum for Wales guidelines on the Expressive Arts area of learning clearly states the importance of widening access to expressive arts (art, dance, drama, film, digital media, music) due to their ability to ‘engage learners physically, socially and emotionally, nurturing their well-being, self-esteem and resilience’. According to the Curriculum for Wales (2019) Expressive Arts guidelines aims to help young people ‘become healthy, confident individuals, ready to lead fulfilling lives as valued members of society’. This emphasis on the arts as well as the broadening of curriculum subjects intends to provide schools with more incentives to provide their pupils with access to theatre. The new curriculum could also offer the four existing contemporary theatre companies for children and young people more opportunities and flexibility to engage young people with theatre and the curriculum. Increased emphasis on the Welsh language in all streams of education in Wales could also mean that the theatre companies will be a means of providing breathing spaces for engagement with the Welsh language.

## Overview of the four Welsh TIE companies

The Arts Council of Wales^4^ is the main funder of the four Welsh theatre for children and young people companies, meaning that most performances are free at point of entry, true to the ethos of the original TIE companies. The National Lottery^5^ also provides additional funding as well as the funding from local councils in the areas that each theatre company serve and, in some cases, small contributions from the schools themselves. The Arts Council of Wales also offer financial support to schools with schemes such as ‘Go and See’ in order to ensure that young people across Wales can access the arts, whatever their socio-economic backgrounds. Some plays aimed at young audiences are also open to the general public, where audience members are expected to pay for their tickets.

All four Welsh theatre companies for children and teenagers, Arad Goch, Cwmni Theatr y Frân Wen, Theatr na nÓg and Theatr Iolo, produce work in both Welsh and English. The linguistic provision of each company to a large extent reflects the linguistic landscape in the areas in which they work. For example, with Cwmni Theatr y Frân Wen, based in Gwynedd, the county with the highest percentage of Welsh speakers, work predominantly through the medium of Welsh (Welsh Government 2022a). Arad Goch may have the most diverse area to cover in terms of bilingualism: on the one hand, they cover Carmarthenshire (39.9% Welsh-speaking) and Ceredigion (45.3% Welsh-speaking); and on the other hand, they also cater for Powys, a county which borders with England where 16.4% of the population speak the Welsh language (2022a). Arad Goch note that Welsh is the company’s working language and that it can perform plays in either language (Arad Goch n.d.). Like Arad Goch, Theatr na nÓg based in Neath, in south Wales, produce each of their productions in both Welsh and English. Indeed, their English productions include some incidental Welsh, for the English-medium schools where pupils will be less confident Welsh speakers. The company takes this approach as they believe that theatre rooted in Wales, be it in Welsh or in English, can contribute to strengthening Welsh identity amongst young people (Turner 2005b, 35). Theatr Iolo, based in Cardiff, adopt a similar approach, although not all productions are performed in both languages. The fact that all four of the remaining companies for young audiences perform in both Welsh and English suggests that the service is valued by schools and local authorities alike, by providing opportunities for engagement with the Welsh language and the content of the Welsh school curriculum.

Between them, the four theatre companies produce over a dozen new plays a year to over 65,000 children and teenagers^6^ in schools, village halls and community centres as well as local and in some cases national theatres. Cwmni Theatr y Frân Wen stage three productions a year on average and although their productions are aimed at teenagers, they are popular with adult audiences too, while the other three companies perform to both children and teenagers. Arad Goch, which produce up to five plays annually, aim to produce a piece for each school Key Stage^7^ within the curriculum. Theatr Iolo produce three plays every year, with one aimed specifically at teenagers, while Theatr na nÓg produce two large scale theatrical projects a year, and although they note that children are their target audience, they produce at least one piece for teenagers every year. In order to provide an insight into one example of drama produced by these companies, the final part of the article will focus on the latest production aimed at teenagers by Arad Goch.

## Case study: Croesi’r Llinell (2022) – Cwmni Theatr Arad Goch

Welsh-language theatre for young audiences has adapted and evolved since the early days of TIE in Wales. The most recent theatre productions (in 2022 and early 2023) explore universal themes which many Welsh or bilingual youths can relate to – especially those from minority or minoritised language contexts – while also being strongly rooted in a specific community.

*Croesi’r Llinell* (trans. ‘County Lines’) is a 2022 play by young playwright Mared Llywelyn (2022) and produced by Arad Goch. *Croesi’r Llinell* is a play in which the lines between online gaming and the real life of a teenager become blurred. Llywelyn’s play is an example of a specifically devised piece which uses powerful theatre as a means of educating teenagers: the play is the result of a collaboration between Arad Goch and Dyfed Powys Police. Dyfed Powys Police asked Arad Goch to create a piece in response to issues relating to county lines the force had been dealing with. County lines is a national issue where organised crime gangs from cities such as London, Birmingham or Liverpool target teenagers and vulnerable adults by manipulating them into carrying and selling drugs. Teenagers themselves were also invited to be part of the creative process by offering their feedback and asking questions in response to five scenes performed in a forum style theatre performance in the early development phase of the play. Youth workers were at hand during the sessions with the teenagers to respond to any concerns or to help any young people that were affected by any of the issues explored in the scenes performed (Turner 2023d). This collaboration is therefore an example of a TIE company responding to a serious issue faced by the young people, local authorities and police force in question. Collaboration, according to Ogden (1997, 51) was one of the main reasons for the success of Welsh TIE companies. *Croesi’r Llinell* demonstrates that collaboration is still at the core of educational theatre for young audiences. Llywelyn’s play has been performed in several secondary schools in Ceredigion, Powys and Carmarthenshire.

The play’s title alludes to several literal and metaphorical crossings within the play for the main character Tal, a 15-year-old boy from north Wales – from crossing the line between the seemingly innocent fantasy world of online gaming to being lured into the perilous world of drugs by a ‘friend’ he meets in the gaming world. During the course of the play, Tal is drawn into crossing the line between the innocence of youth and online gaming to crossing literal trainlines as a ‘runner’, who gets sent across county boundaries from large cities such as London or Liverpool to areas such as Llanelli, Newton and Haverfordwest to deliver and sometimes to sell Class A drugs for a drug dealer at the other end of the trainline. The play clearly demonstrates that living in semi-rural areas in Wales doesn’t make youths immune to the influence of these county lines, nor does being behind the screen of a computer. The power of exploring such issues through the medium of Welsh also shows that such issues are closer to home than expected. Language, technology and healthy relationships are among the themes explored in the play. Questions in relation to authority or parents and teachers as well as emotional wellbeing are also raised in *Croesi’r Llinell* with the aim of starting a dialogue around these themes.

The play’s link with the Curriculum for Wales is clearly outlined in the educational pack produced. The educational pack includes suggested activities to complement different areas of the new Curriculum for Wales such as Expressive Arts, Languages, Literacy and Communication, Health and Wellbeing. Activities based on the play in the form of pieces of dialogue to analyse and questions to explore with the aim of getting young people to engage with the issues explored in the production in Welsh are also provided. Academics such as Helen Nicholson (2005, 24) have emphasised the power of theatre as it can be used as a mirror on the young audiences’ lives and in encouraging and inviting that audience to contemplate their response to the situations acted out in front of them. One could argue that doing so in a minority language, especially when exploring a challenging theme, adds another dynamic to the performance for young Welsh-speaking audiences as it means that language can be used beyond the confines of the classroom to discuss emotive and sometimes taboo issues.

The play adopts both north Wales and south Wales dialect as well as both official languages of Wales, Welsh and English. Tal and his friend Cian speak in northwest Wales dialect while Nat, who is originally from south Wales speaks in south Wales dialect which ensures that young people from both north and south Wales feel a connection to the character’s language. English is the language used by the county lines drug dealer, which could possibly make the play more realistic to young people, as many of the drug dealers are based in large English cities. The young audience are encouraged to engage with questions of language in the educational pack and it quotes directly form the Curriculum for Wales with specific questions relating the use of varying register and dialect and to language’s role in developing cultural identity and belonging (Cwmni Theatr Arad Goch 2022).

This is just one recent example of a piece of theatre produced to engage teenagers with theatre and the Welsh language by one of the four Welsh theatre companies in question. Other recent examples include Bethan Marlow’s stage adaptation of Alys Conran’s novels *Pijin/Pigeon*, produced by Theatr Iolo, Theatr Genedlaethol Cymru^8^ and Pontio (Marlow 2023) in which the strength and resilience of a formative childhood friendship between the main character Pijin and his best friend Iola after a series of traumatic events are explored. The trauma of those events is felt in every aspect of the characters’ lives, from their relationship with each other, the Welsh language, memory, place and coming of age. Coming of age, language and belonging are also at the heart of *Ynys Alys* [Alys’ Island] (Evans-Jones 2022) by Cwmni’r Frân Wen, a production in which ‘[t]heater, pop music and rap collide’ (Cwmni’r Frân Wen 2022). The collision of forms is used to express the main character’s linguistic and cultural hybridity. *Ynys Alys*’s script, written by Gareth Evans-Jones is based on a series of workshops with Welsh-speaking teenagers on issues which were important to them such as the themes mentioned above, while the music was a collaboration between Casi Wyn and Lemarl Freckleton. Social class, justice and language are among the main themes of *Prawf Elgan Jones* or *The Trial of Elgan Jones* in English (2022) in Theatr na nÓg’s latest production for young audiences, written by Geinor Styles (2022), the Artistic Director of Theatr na nÓg. Young audience members are transported back to 1898 in *Prawf Elgan Jones* and are called upon to carry out jury service in the trial of Elgan Jones, a 12-year-old boy who is accused of poaching, stealing and killing the gamekeeper of a country estate in South Wales. The play introduces the audiences to the legal and socio-economic context of the era as well as exploring social class and complex attitudes towards the Welsh language at the turn of the twentieth century. All four of the plays mentioned in this article explore challenging questions in relation to language and identities in dynamic and creative ways and by doing so, encourage their audiences to do the same.

## Youth involvement with each of the companies

As well as creating breathing spaces in the form of performances and associated educational activities, all four companies host a wide variety of initiatives aimed at encouraging youth engagement with the theatre and theatre through the medium of Welsh in most cases. Cwmni’r Frân Wen, Arad Goch and Theatr na nÓg are most certainly leading the way in terms of engaging young people with both theatre and the Welsh language. Other than a Young Playwright competition, Theatr Iolo are not as active on this front.

Some of the initiatives aimed specifically at teenagers include drama clubs such as Arad Goch’s weekly drama club, AGwedd (translated as ‘attitude’) and Cwmni’r Fran Wen’s Youth theatre group. As well as using theatre groups as a means of encouraging teenagers to engage with theatre through the medium of Welsh, several of the initiatives offered by the theatre companies for young audiences offer young people the space and language to explore and express questions in relation to well-being, language and identity. Schemes include as ‘Fi Di Fi’ (translation, ‘I am who I am’) run by Cwmni’r Frân Wen which offers teenagers the opportunity to work with a team of multimedia artists led by Cwmni’r Frân Wen to explore well-being through the arts in Welsh and Theatr na nÓg’s Erasmus + scheme entitled ‘Unpacking Feelings’. The ‘Unpacking Feelings’ project is an expressive arts project devised in response to some of the mental health challenges many young people are facing as a result of the COVID-19 pandemic. Expressing such sensitive emotions are challenging and the challenge is heightened for those youths from English-speaking families who attend Welsh-medium education. Theatr na nÓg have developed a series of performance workshops along with creative arts experts and teaching professionals in Spain, Malta, Cyprus with the aim of encouraging and facilitating expressing those feelings in a non-direct way through drama games, mirroring and script work in their second language, Welsh, in the case of many of the youths attending the schools involved in Swansea.

Despite not being an exhaustive list of all the activities and schemes run by Welsh theatre companies for young audiences the above does offer an overview of how the companies create dynamic breathing spaces for youth engagement with the Welsh language and questions of identity and belonging through the medium of theatre beyond the productions themselves.

## Conclusion

On the one hand, existing between several worlds – between Welsh and English languages, and between education and theatre, and navigating the sometimes contradictory policies of both – could be considered a challenge for Welsh-language TIE. However, this in-between space provides exciting opportunities for a number of reasons. Firstly, teenagers themselves often occupy this in-between space, between being seen as children on the one hand and adults on the other. The tensions a teenager from a minoritised language context may feel are even greater as they feel torn between languages, cultures and identities, as demonstrated in the themes explored and the mediums and languages used in all the plays mentioned in this article. This in-between space offers rich opportunities for collaboration, innovation and creativity.

With the increased emphasis on the expressive arts and the Welsh Government’s target of reaching a million Welsh speakers by 2050, creating breathing spaces such as those carved out by all four companies aimed at children and youths will become increasingly important. Due to a drop in the number of Welsh speakers and the Welsh Government’s language strategy to reach a million Welsh speakers by 2050, the role of the education system in Wales in transferring the Welsh language to the next generation is likely to intensify. Therefore, creating breathing spaces via a theatre network which invites youths to imagine themselves in plausible and challenging situations beyond the education system through the medium of Welsh is a very valuable resource indeed, one which other minority or minoritised language contexts could implement.

## Notes

1 Created in 1965, The Welsh Office, headed by a Secretary of State for Wales, was a department responsible for Wales within the Government of the United Kingdom. Since the beginning of Welsh devolution in 1999, the nature of the role has changed as many of the powers of the Welsh Office now lie in the Senedd. The role of Secretary of State for Wales still exists but they are now responsible for running the Wales Office which aims to facilitate the devolution settlement and representing Welsh interests in Westminster.

2 Since 1996 Arad Goch also host the Agor Drysau/Opening Doors festival, Wales’ only international theatre festival for the performing arts for young audiences. Agor Drysau/Opening Doors was started with the aim of providing Welsh performers with the opportunity to showcase their work to an international audience as well as attracting international theatre companies for young audiences to Wales.

3 There are currently 3 school categories in Wales: Welsh-medium education where every subject other than English is taught in Welsh, bilingual education where some subjects are taught in Welsh and English-medium education where Welsh is taught as a second language and every subject other that Welsh is taught through the medium of English.

4 According to the Arts Council of Wales’ 2021–2022 Annual review the companies received the following sums of funding: Cwmni Theatr Arad Goch: £350,703, Theatr na nÓg: £319,569, Theatr Iolo: £259,573, Cwmni’r Frân Wen: £235,573. Arts Council of Wales. 2022. General Activities and Lottery Distribution: Report and Financial Statements for the Year Ended 31 March 2022. Accessed 30 January 2023. https://arts.wales/about-us/accountability/annual-reports

5 This funding isn’t as consistent at the Arts Council of Wales Funding and it is usually to support individual projects such as Young Ambassador Project – Project | The National Lottery Community Fund (tnlcommunityfund.org.uk)

6 Arad Goch state that they perform to over 24,000 children and teenagers every year (Arad Goch n.d.), Theatr na nÓg perform to 16,000 young people across south Wales (Theatr na nOg n.d.) while Theatr Iolo performed to 5465 in 2022 (Theatr Iolo 2022) and Cwmni’r Frân Wen performed to 19,668 people in 2022.

7 Key Stages outline the expected level of knowledge of each key period in the curriculum in Wales, England and Northern Ireland. There are 5 key stages in total, KS1 –5- to 7-year-olds, KS2 – 7–11 year olds, KS3 – 11–14 year olds and KS4 – 14–16 year olds and KS5 more commonly referred to as college or sixth form 16 to 18.

8 Theatr Genedlaethol Cymru, established in 2003 is the Welsh language national theatre of Wales.
